# A combination SMS and transportation reimbursement intervention to improve HIV care following abnormal CD4 test results in rural Uganda: a prospective observational cohort study

**DOI:** 10.1186/s12916-015-0397-1

**Published:** 2015-07-06

**Authors:** Mark J. Siedner, Data Santorino, Alexander J. Lankowski, Michael Kanyesigye, Mwebesa B. Bwana, Jessica E. Haberer, David R. Bangsberg

**Affiliations:** Division of Infectious Diseases, Department of Medicine, Massachusetts General Hospital and Harvard Medical School, 100 Cambridge Street, 15th Floor, Boston, MA 02114 USA; Mbarara University of Science and Technology, Mbarara, Uganda; Department of Medicine, Massachusetts General Hospital and Harvard Medical School, Boston, MA 02114 USA; Department of Medicine, Massachusetts General Hospital, Harvard Medical School, and Harvard School of Public Health, Boston, MA 02114 USA

**Keywords:** HIV/AIDS, Sub-Saharan Africa, Clinical trial, Short message service, Financial incentive, Antiretroviral therapy

## Abstract

**Background:**

Up to 50 % of HIV-infected persons in sub-Saharan Africa are lost from care between HIV diagnosis and antiretroviral therapy (ART) initiation. Structural barriers, including cost of transportation to clinic and poor communication systems, are major contributors.

**Methods:**

We conducted a prospective, pragmatic, before-and-after clinical trial to evaluate a combination mobile health and transportation reimbursement intervention to improve care at a publicly operated HIV clinic in Uganda. Patients undergoing CD4 count testing were enrolled, and clinicians selected a result threshold that would prompt early return for ART initiation or further care. Participants enrolled in the pre-intervention period (January – August 2012) served as a control group. Participants in the intervention period (September 2012 – November 2013) were randomized to receive daily short message service (SMS) messages for up to seven days in one of three formats: 1) messages reporting an abnormal result directly, 2) personal identification number-protected messages reporting an abnormal result, or 3) messages reading “ABCDEFG” to confidentially convey an abnormal result. Participants returning within seven days of their first message received transportation reimbursements (about $6USD). Our primary outcomes of interest were time to return to clinic and time to ART initiation.

**Results:**

There were 45 participants in the pre-intervention period and 138 participants in the intervention period (46, 49, and 43 in the direct, PIN, and coded groups, respectively) with low CD4 count results. Median time to clinic return was 33 days (IQR 11–49) in the pre-intervention period and 6 days (IQR 3–16) in the intervention period (*P* < 0.001); and median time to ART initiation was 47 days (IQR 11–75) versus 12 days (IQR 5–19), (*P* < 0.001). In multivariable models, participants in the intervention period had earlier return to clinic (AHR 2.32, 95 %CI 1.53 to 3.51) and earlier time to ART initiation (AHR 2.27, 95 %CI 1.38 to 3.72). All three randomized message formats improved time to return to clinic and time to ART initiation (*P* < 0.01 for all comparisons versus the pre-intervention period).

**Conclusions:**

A combination of an SMS laboratory result communication system and transportation reimbursements significantly decreased time to clinic return and time to ART initiation after abnormal CD4 test results.

**Trial registrations:**

Clinicaltrials.gov NCT01579214, approved 13 April 2012.

**Electronic supplementary material:**

The online version of this article (doi:10.1186/s12916-015-0397-1) contains supplementary material, which is available to authorized users.

## Background

Despite expanded access to antiretroviral therapy (ART) [1], programs in resource-limited settings continue to document high mortality rates during early stages of disease [2–4]. An important contributor to poor outcomes is suboptimal retention of patients between HIV diagnosis and ART initiation [5], when mortality rates are highest [6, 7], and approximately 20–50 % of patients are lost to care [8–10].

Structural barriers to care in resource-limited settings, including transportation costs and absence of communication between providers and patients, are major contributors to poor linkage [11–14]. Communication of critical clinical information to patients in the community represents a particular challenge. For example, reporting and responding to abnormal clinical investigations typically requires patients to return for a repeat clinical visit, which comes at significant cost and time away from economic activity for patients [15]. In cases of an indication for ART initiation, treatment failure, severe treatment complications, or evidence of opportunistic infection, such reporting and intervention delays result in adverse outcomes and/or compromise future treatment options.

Scalable interventions that mitigate structural barriers to clinical care in resource-limited settings are urgently needed. Mobile health (mHealth) applications hold promise in this area by leveraging existing cellular phone infrastructure to improve patient-provider communication and prioritize care delivery for those most in need. Cellular phone coverage in sub-Saharan Africa increased from 5 to 70 % of the population during the past decade, while personal subscriptions increased from 16 to 380 million [16, 17]. While short message service (SMS) reminders have been shown to improve ART adherence [18, 19], there has been limited data to evaluate the efficacy of mHealth interventions to improve clinical care. We previously reported results of a survey to assess the acceptability of an SMS-based laboratory results notification system to communicate abnormal laboratory results to patients at a publicly operated HIV clinic in rural, southwestern Uganda [20]. We found that acceptance was nearly 100 % and that benefits of improved patient-provider communication outweighed potential concerns about breaches of confidentiality. We now report results of a follow-up intervention trial to evaluate an mHealth laboratory result notification system coupled with transportation stipends to improve care for people living with HIV undergoing critical laboratory tests in rural Uganda. We hypothesized that the mHealth application coupled with transportation reimbursements would reduce time to clinic return and time to ART initiation for patients with low CD4 count results.

## Methods

### Study population and eligibility criteria

Study participants were enrolled from a publicly operated President’s Emergency Plan for HIV/AIDS Relief (PEPFAR)-supported HIV clinic at the Mbarara Regional Referral Hospital in Mbarara, Uganda. Eligibility criteria included: a) current enrollment at the adult HIV clinic, b) self-reported access to a cellular phone, whether personal or shared, c) residence in a district immediately surrounding the clinic (Ibanda, Isingiro, Kirihura, Mbarara, or Ntungamo Districts), and d) undergoing a CD4+ T-lymphocyte (CD4) test determined by the ordering clinician to be of critical importance (that is, a test for which an abnormally low result would ideally prompt early return to care). Clinicians selected the abnormal CD4 result threshold on the day the laboratory test was ordered. The threshold was meant to specify a result below which an early return to clinic would be requested for clinical evaluation. Potential participants could be ART-naïve or on ART (with concern for treatment failure). We did not include viral load testing as an inclusion criterion because it is not available free of charge at the clinic and was rarely performed outside of research activities.

### Intervention development

The intervention was developed based on a conceptual framework developed through pre-study mixed-methods research. Pre-intervention interviews with patients and providers at the clinic revealed two major barriers to clinic return after abnormal test results at this site: 1) lack of communication between staff and patients outside of clinical visits, which limited patient decision-making capacity [20], and 2) inadequate access to financial support for costs of transportation to clinic [14, 15, 21]. Based on this empirical evidence, we hypothesized that an effective intervention to improve response to abnormal test results would require both improved clinical communication to patients and financial reimbursement to overcome economic barriers to care. After the basic features of the intervention were selected, we implemented a co-creation model involving researchers, programmers, clinicians, and patients in key considerations about the SMS intervention [22], including 1) optimizing message formats to maximize confidentiality and privacy, 2) selection of the frequency, duration, and timing of messages, 3) language preferences, and 4) inclusion of an option to respond to messages for confirmation of receipt. We pilot tested the system with both study staff and clinicians to optimize interpretation, readability, and ease of use.

### Pre-intervention study group and procedures

We initially considered a fully randomized control trial, including a control group without a notification SMS message. We decided against this for two reasons. First, in a preliminary acceptability study at the clinic, we found that 100 % of surveyed participants desired cellular phone communication about their laboratory test results [20]. Second, ethical concerns were raised about the safety of a control arm in which participants who do not receive SMS messages could misinterpret lack of an SMS message as positive information about their test results. Thus, in place of a fully randomized trial design, we prospectively collected data on eligible participants in two stages: 1) a pre-intervention period (January – August 2012) and 2) an intervention stage (September 2012 – November 2013), as has been done with a similar clinical intervention targeting abnormal CD4 count results [23]. During the pre-intervention stage, clinicians completed eligibility forms for each participant, including confirmation of access to a cellular phone, district of residence, and selection of the abnormal result threshold for the CD4 test, which would prompt a request for an early return to clinic. Standard clinical forms were completed to collect data on sociodemographic and clinical characteristics. We also collected data on the laboratory result and result date, time from laboratory result to clinic return, and for ART-naïve participants, time to ART initiation.

### Intervention study group and procedures

During the intervention period, clinicians used eligibility criteria identical to those used during the pre-intervention period, including selection of an abnormal CD4 count threshold to trigger the SMS and transportation reimbursement intervention. We discussed message design with study clinicians during the intervention design phase. They requested an individualized determination of abnormal test results over a standardized method, largely to allow enrollment of patients already on therapy who were considered to be at risk for treatment failure. Similarly, clinicians scheduled the next clinic appointment prior to study enrollment during both study periods. Eligible participants completed written informed consent to receive health messages on their phone, completed a baseline structured survey to collect SMS scheduling preferences, and were given a brief instructional session to describe possible messages and select a personal identification number (PIN) to open messages.

Those with a CD4 test result above the clinician-specified threshold (that is, a “normal” laboratory result) received a single SMS message stating that their test result was within the normal range and that they should return to clinic as scheduled. For participants with a CD4 test result below the stated threshold (an abnormal result), study staff used the randomization module within the Research Electronic Data Capture (REDCap [24]) study database to assign participants to an intervention arm. Participants were randomized in a 1:1:1 design to receive one of the following three SMS message formats: 1) an unprotected SMS indicating an abnormal test result and that they should return to clinic as soon as possible: “This is an important message from your doctor. You had an abnormal test result. You should return to clinic as soon as possible.” (direct message). This message was meant to maximize clarity. 2) The use of a PIN-protected SMS message, which displayed an identical message as the direct message only after successful entry of the correct PIN code (PIN message). The PIN code was intended to augment message privacy. 3) The use of a message reading “ABCDEFG” (coded message). This message was coded without mention of clinical information to maximize confidentiality, but not require the participant to remember and enter a PIN code. Research assistants explained to participants on enrollment that the message indicated an abnormal test result that should prompt early return to clinic.

Messages were scheduled and initiated with CommCare (Dimagi, Inc., Cambridge, MA, USA), a web-based mobile health application. Participants could receive up to seven daily messages at preferred days of the week and times of day (6AM, 9AM, 5PM, or 9PM) that were selected by participants during the baseline survey. On the date and time of each scheduled message, CommCare sent a web-based automated prompt with the content of the message to a Ugandan-based telecommunications company (Yo! Voice Solutions and Software Development, Kampala, Uganda), which relayed an automated SMS to the indicated phone number. The messages cost $0.02 each to send, and were paid for by the study. Participants in the intervention period with a normal laboratory result received a single message at the next preferred date and time that was indicated on their enrollment scheduling form. Participants in the intervention group received a single abnormal result message daily, up to a maximum of seven days after the abnormal result. Participants who returned to clinic within seven days of the first abnormal result message received a transportation reimbursement stipend of 15,000 Ugandan shillings (approximately 6$USD, the average estimated cost for transportation to clinic in the clinic catchment area). Participants with normal laboratory results or those presenting after seven days from the first message were not eligible for transportation reimbursement. There were no blinding procedures as part of this study.

For both study groups, we recorded the date of clinic return after an SMS message and, for the ART-naïve participants, the date of ART initiation. To assess receipt and comprehension of the messages, research assistants called participants in the intervention period who did not return to clinic within 14 days (after determination of primary outcome success or failure). We performed home-based tracking for participants with abnormal laboratory results who did not return to clinic by 28 days.

### Sample size determination and statistical analysis

In our initial protocol, our prespecified primary outcome of interest was return to clinic within 7 days of an abnormal test result notification. However, we noted during study planning that the time from test result to first SMS transmission varied by participant. For example, if a participant selected only a single day to receive messages (for example, Mondays only) and the laboratory result returned on the following calendar day (Tuesday), the maximum time period from laboratory result to receipt of the first message was 7 days, which corresponded to a maximum period of 14 days from test result to clinic return to achieve the desired outcome. To ensure unbiased outcome assessment between study arms, we changed our primary outcome to: return to clinic within 14 days of when the laboratory result was received back at the clinic from the laboratory. The study was powered to detect a difference in return time for participants with abnormal results. Whereas the study was initially powered based on assumptions about clinic return with 7 days, we updated our sample size estimates with the 14-day outcome using data from the pre-intervention period. During the pre-intervention period, approximately 30 % of 45 participants with abnormal laboratory results returned to clinic within 14 days. We planned to enroll 45 participants with abnormal laboratory results in each intervention arm in order to have 80 % power to detect a doubling in return rate (60 % clinic return rate within 14 days of abnormal laboratory result) between the pre-intervention period and each of the intervention groups.

We conducted survival analyses and fit Cox proportional hazards regression models to estimate differences between study arms in: 1) time to return to clinic after an abnormal result and 2) time to initiation of ART among ART-naïve participants. Our primary predictors of interest were message type (comparing each of the three message formats with the control group) and study period (comparing the control period with the intervention period). Models were adjusted for known predictors of clinic retention in care based on prior studies, including age, gender, district of residence, education, and CD4 result [25]. Participants in the intervention period who were tracked at home were allocated as treatment failures for all study endpoints. For time-to-event analyses, we right censored participants at 180 days who did not return to clinic by that time. Finally, to estimate the impact of the intervention for those with normal laboratory results, we also compared the proportion of participants with normal results who returned to clinic on or within 7 days of their scheduled return visit date. All statistical analyses were performed with Stata Version 13 (StataCorp, College Station, TX, USA).

### Ethical review

The study was reviewed and approved by the ethical review committees of the Mbarara University of Science and Technology (Reference Number: 1/7), Partners Healthcare (Partners Reference Number: 2011P001538), and the Ugandan National Council of Science and Technology (Reference Number: IS 83). The trial and study protocol were registered prior to study procedures at clinicaltrials.gov (NCT01579214).

## Results

**Fig. 1:**
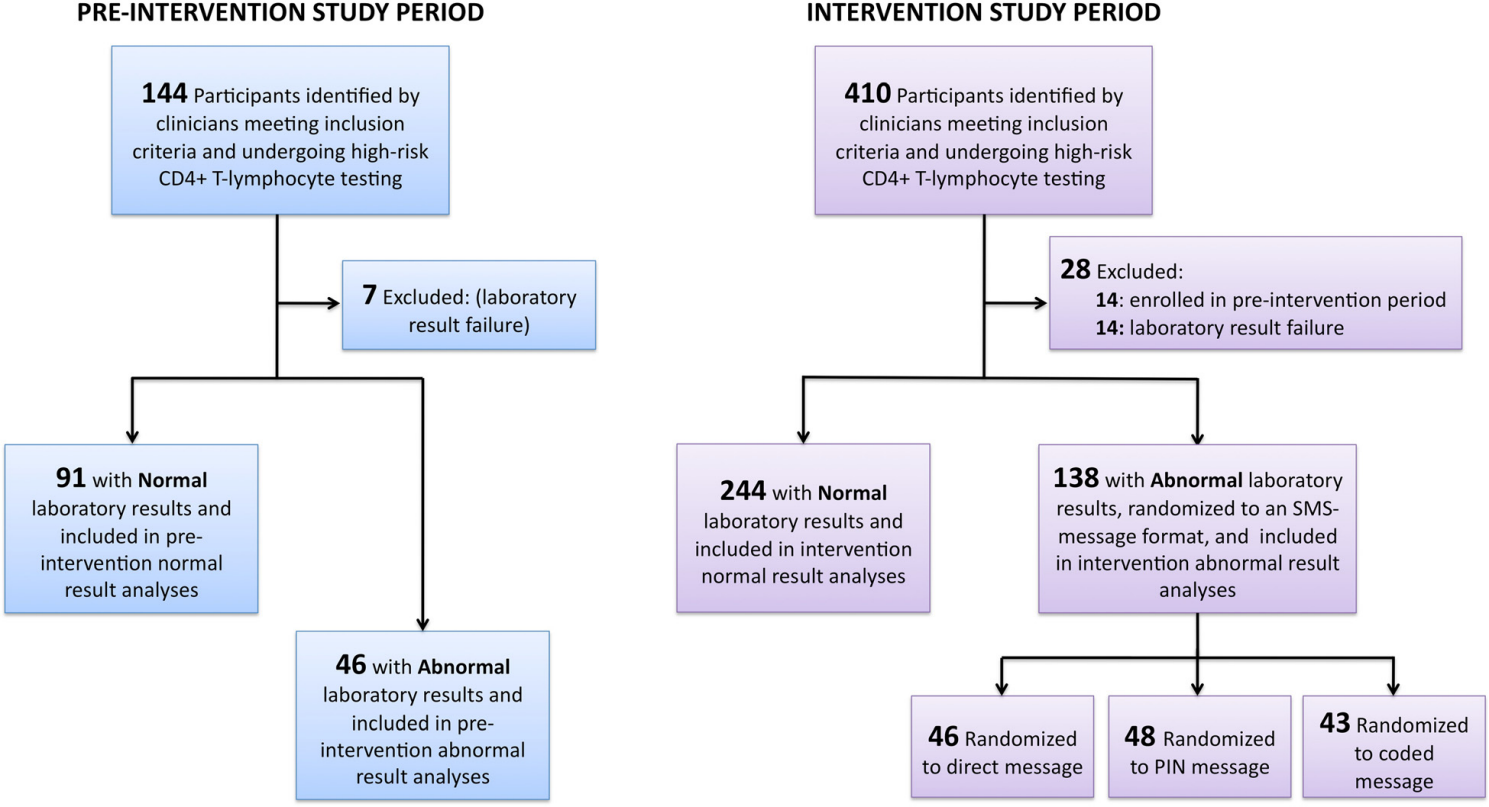
Study flowchart for a combination intervention to improve HIV linkage to care in rural Uganda. Participants in the pre-intervention period served as a control group. Participants in the intervention period with CD4 count below a clinician-selected threshold were randomized to receive one of three short message service (SMS) text messages to inform them of abnormal laboratory results: 1) a direct message which stated that test results were abnormal and they should return to clinic, 2) a personal identification number (PIN)-protected message that was otherwise identical to the direct message, and 3) a coded message reading “ABCDEFG” to deliver an abnormal result message confidentially. Those who returned to clinic within seven days received a transportation incentive

Of 554 participants screened, 21 (6 %) were excluded due to invalid laboratory results (n = 21). An additional 2 % (n = 12) were excluded from the intervention period due to prior participation in the control period (Fig. 1). Of the remaining 521 participants enrolled, 183 (35 %) had abnormal CD4 results. There were 45 participants in the pre-intervention period and 138 participants in the post-intervention period (46, 49, and 43 participants randomized to the direct, PIN, and coded message arms, respectively). Characteristics of participants with abnormal laboratory results are described in Table 1. Participants in the pre-intervention period with abnormal results had a higher median age and fewer were ART-naïve at the time of enrollment. There were no meaningful differences between participants in each of the three post-intervention study groups (Additional file 1: Table S1). The number of participants who were tracked at home at study day 28 (and allocated as failures to initiate ART) was 2 (4 %), 3 (6 %), and 1 (2 %), respectively, in the direct, PIN, and coded arms.

**Table 1:** Characteristics of study participants with abnormal CD4+ T-lymphocyte results

	Pre-intervention period	Intervention period	*P*-value
	(No SMS message)	(SMS + transportation reimbursement)	
	(n = 45)	(n = 138)	
Female gender (n, %)	19 (42)	75 (54)	0.16
Age (median, IQR)	38 (31 – 44)	30 (25 – 38)	<0.001
Education (n, %)			0.71
<Primary	3 (8)	12 (9)	
Any primary	16 (42)	71 (51)	
Any secondary	12 (32)	37 (27)	
>Secondary	7 (18)	18 (13)	
ART-naïve (n, %)	26 (58)	110 (80)	<0.001
Mbarara resident (n, %)	32 (71)	83 (60)	0.19
Days from enrollment until laboratory result, median (IQR)	11 (8 – 19)	10 (8-15)	0.32
Clinician-specified abnormal CD4 result threshold, median (IQR)	350 (250 – 350)	350 (350 – 350)	<0.001
CD4 result, median (IQR)	185 (106 – 262)	225 (110 – 293)	0.29

SMS: short message service text message

PIN: personal identification number

IQR: interquartile range

ART: antiretroviral therapy

**Fig. 2:**
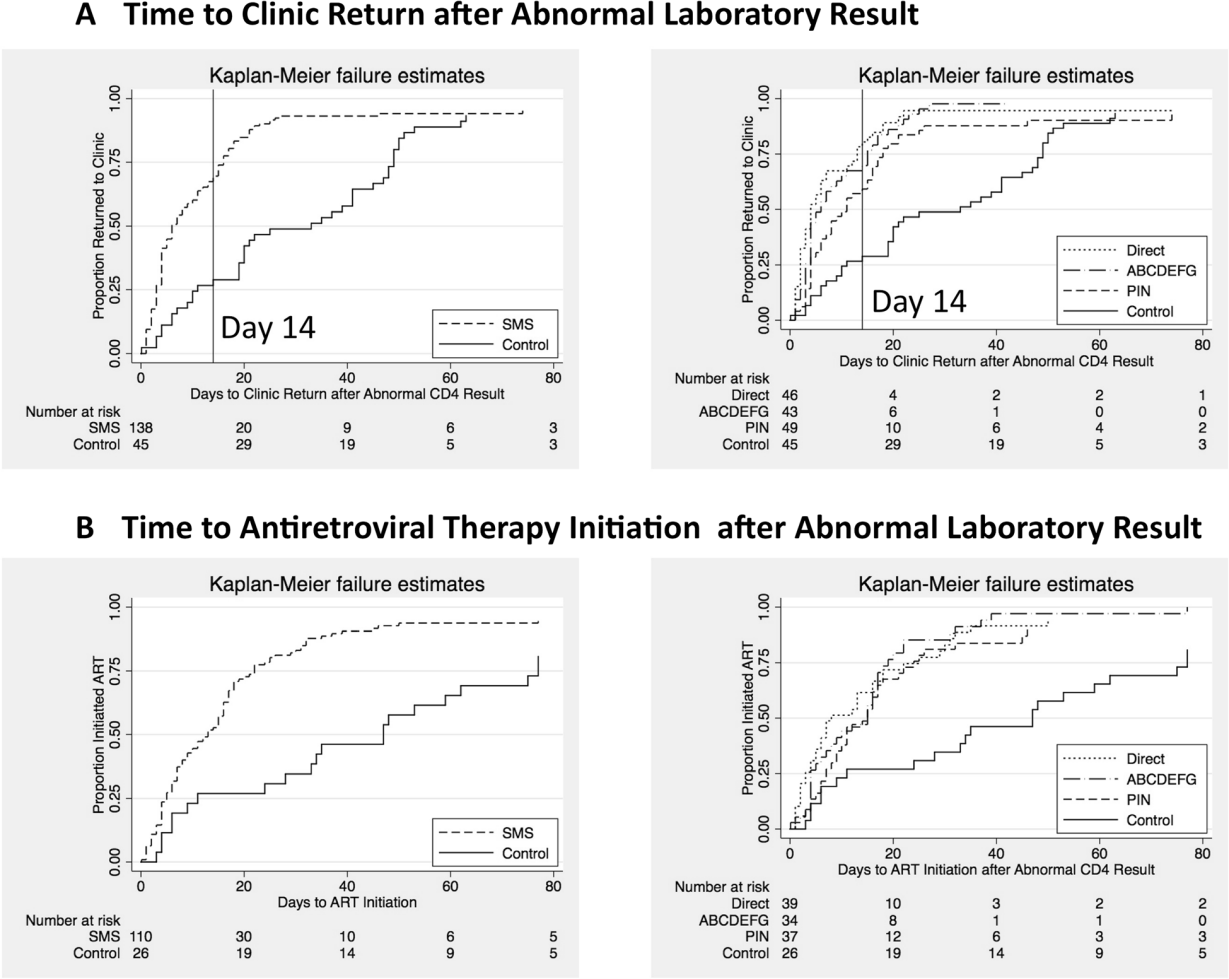
Kaplan-Meier plots demonstrating days from abnormal CD4 count result until return to clinic (**a**) and days from abnormal CD4 count result until ART initiation (**b**). The left panels compare results for participants in the pre-intervention period (control) versus all participants in the intervention period (SMS). The right panels compare results between the pre-intervention period (control) and each of the three randomized SMS message groups (direct, PIN, and coded)

The proportion of participants returning with 14 days, the primary outcome of interest, was 27 % (12/45) in the pre-intervention period and 67 % (93/138) in the intervention period (*P* < 0.001, Table 2). Median time to clinic return was 33 days (IQR 11–49) in the pre-intervention period and 6 days (IQR 3–16) in the intervention period, whereas median time to ART initiation decreased from 47 days (IQR 11–77) to 13 days (5–22 days). In multivariable models adjusted for age, gender, district of residence, educational attainment, and CD4 result, participants in the intervention period had shorter time to clinic return (adjusted hazard ratio [AHR] = 2.32, 95 %CI 1.53–3.51, *P* < 0.001, Table 3a, Fig. 2a) and shorter time to ART initiation (AHR = 2.26, 95 %CI 1.38–3.72, *P* = 0.001, Table 3b, Fig. 2b).

**Table 2:** Crude outcomes by study group

Time to clinic return	Pre-intervention period (No SMS message)	Intervention period (SMS + transportation reimbursement)		Direct SMS message group	PIN SMS message group	Coded SMS message group
	(n = 45)	(n = 138)	*P*-value*	(n = 46)	(n = 49)	(n = 43)
Days from abnormal laboratory result to clinic return, median (IQR)	33 (11 – 49)	6 (3 – 16)	<0.001	4 (2 – 13)	11 (4 – 18)	6 (3 – 15)
Proportion returned within 14 days of abnormal result (n, %)	12/45 (27)	93/138 (67)	<0.001	36/46 (78)	28/49 (57)	29 /43(67)
Proportion returned within 28 days of abnormal result (n, %)	22/45 (49)	128/138 (93)	<0.001	43/46 (94)	43/49 (89)	42/43 (98)
Proportion returned before scheduled visit (n, %)	11/45 (24)	96/137 (70)	<0.001	33/46 (72)	34/49 (69)	29 /42 (69)
Time to ART initiation (ART-naïve only)	Pre-intervention period (No SMS message)	Intervention period (SMS + transportation reimbursement)	*P*-value*	Direct SMS message group	PIN SMS message group	Coded SMS message group
	(n = 45)	(n = 138)		(n = 37)	(n = 39)	(n = 34)
Days from abnormal laboratory result to ART initiation, median (IQR)	47 (11 – 77)	13 (5 – 22)	<0.001	8 (3 – 25)	15 (7 – 24)	15 (4 – 19)
Participants initiating ART within 14 days of abnormal result (n, %)	7/26 (27)	57/110 (52)	0.06	24 (62)	17 (46)	16 (47)
Participants initiating ART within 28 days of abnormal result (n, %)	8/26 (31)	89/110 (81%)	<0.001	30/39 (77)	30/37 (81)	29/34 (85)

*P-values for comparisons between the pre-intervention and post-intervention periods, using log-rank testing for continuous time to return outcomes and chi-squared testing for categorical outcomes

IQR: interquartile range

SMS: short message service text message

ART: antiretroviral therapy

**Table 3:** Univariable and multivariable Cox proportional hazards estimates demonstrating hazard of time to clinic return (A) and time to ART initiation (B)

A. Outcome: Time to return to clinic after an abnormal CD4+ T-lymphocyte result				
	Univariable estimate		Multivariable estimate	
Characteristic	HR (95 %CI)	*P*-value	AHR (95 %CI)	*P*-value
Age (each year)	0.99 (0.98 – 1.01)	0.435	1.02 (1.00 – 1.03)	0.043
Female gender	0.98 (0.73 – 1.32)	0.885	0.94 (0.68 – 1.28)	0.673
Educational attainment (Ref: primary or less)	1.13 (0.84 – 1.54)	0.419	1.00 (0.73 – 1.38)	0.994
Mbarara district resident	1.17 (0.86 – 1.59)	0.323	1.33 (0.96 – 1.85)	0.082
ART-naïve	1.56 (1.10 – 2.21)	0.011	1.48 (1.00 – 2.19)	0.051
CD4 result (each 100 cells)	0.87 (0.75 – 1.01)	0.062	0.80 (0.69 – 0.93)	0.004
Study period				
Control (no message)	REF	--	REF	--
Intervention (any message)	2.17 (1.53 – 3.09)	<0.001	2.32 (1.53 – 3.51)	<0.001
Study group				
Control (no message)	REF	--	REF	--
Direct SMS message	2.73 (1.74 – 4.26)	<0.001	3.05 (1.73 – 5.35)	<0.001
PIN SMS message	1.71 (1.12 – 2.61)	0.013	2.03 (1.20 – 3.45)	0.009
Coded SMS message	3.98 (2.38 – 6.65)	<0.001	3.28 (1.85 – 5.65)	<0.001
B. Outcome: Time to initiation of antiretroviral therapy after an abnormal CD4+ T-lymphocyte result				
	Univariable estimate		Multivariable estimate	
Characteristic	HR (95 %CI)	*P*-value	AHR (95 %CI)	*P*-value
Age (each year)	1.00 (0.98 – 1.02)	0.968	1.02 (1.00 – 1.04)	0.067
Female gender	0.99 (0.70 – 1.40)	0.956	1.12 (0.77 – 1.63)	0.543
Educational attainment (Ref: primary or less)	0.78 (0.55 – 1.11)	0.169	0.71 (0.49 – 1.03)	0.073
Mbarara district resident	0.92 (0.65 – 1.32)	0.664	0.98 (0.68 – 1.44)	0.946
CD4 result (each 100 cells)	0.70 (0.59 – 0.83)	<0.001	0.69 (0.58 – 0.82)	<0.001
Study period				
Control (no message)	REF			
Intervention (any message)	2.21 (1.40 – 3.47)	0.001	2.27 (1.38 – 3.72)	0.001
Study group				
Control (no message)	REF			
Direct SMS message	2.24 (1.31 – 3.84)	0.003	2.40 (1.27 – 4.54)	0.007
PIN SMS message	1.91 (1.11 – 3.27)	0.018	2.36 (1.21 – 4.61)	0.011
Coded SMS message	3.54 (1.94 – 6.47)	<0.001	3.06 (1.61 – 5.84)	0.001

All three message formats outperformed the pre-intervention period. Median days to clinic return after abnormal results was 4, 11, and 6 days in the direct, PIN, and coded message groups, respectively (*P* < 0.010 for all comparisons by log-rank testing versus the control arm); and median time to ART initiation was 8 (*P* = 0.002), 15 (*P* = 0.016), and 15 days (*P* < 0.001). All comparisons between the pre-intervention period and each individual message format remained statistically significant in multivariable analyses adjusted for sociodemographic and clinical factors (Table 3a,b, Fig. 2a,b). There were no significant differences between message groups in the proportion that returned within 14 days (*P* = 0.09), the proportion that returned before their scheduled visits (*P* =0.96), or the proportion that initiated ART within 14 days (*P* = 0.32). Lastly, among those with normal results, a greater proportion who received an SMS message returned within 7 days of their scheduled return date (73 versus 62 %, *P* = 0.044, Additional file 2: Table S2). No participants died during the study and no other adverse events related to the study were reported.

## Discussion

A combination of an SMS-based laboratory results notification system coupled with transportation reimbursements substantially shortened time of return to care and time to ART initiation following abnormal CD4 count results among patients at a publicly operated clinic in rural, southwestern Uganda. While the primary intervention was non-randomized, the effect size was large in all intervention period subgroups, and a benefit was detected independent of CD4 count, age, gender, home district, and educational attainment. These data from a prototypical, government-operated, PEPFAR-supported clinic in a rural, resource-limited setting in sub-Saharan Africa offer a promising strategy to combat widespread patient attrition during early stages of HIV care in the region.

Our study adds to a small list of proposed strategies for improving timely return to HIV care in similar settings. The most promising of these has been adoption of point-of-care CD4 count testing. A study in Mozambique with a similar before-and-after comparative effectiveness design showed an approximately 50 % reduction in loss to follow-up and a comparable decrease in ART initiation time from 48 to 20 days with the use of point-of-care CD4 count testing [23]. However, a second retrospective analysis of the impact of point-of-care testing on ART initiation at a clinic in South Africa failed to demonstrate improved return times in a non-research setting [26]. An important comparative advantage of point-of-care CD4 testing over the current intervention is the ability to streamline ART initiation without the cost or delay associated with additional clinic visits. While not evaluated in our study, similar SMS-based notification programs have previously been shown to include secondary benefits, including the potential to improve patient-staff communication and perceptions of quality of care [27–29]. Preliminary evidence of this effect was suggested by the earlier return times among study participants with abnormal laboratory results, and the higher proportion of participants with normal laboratory results who returned within seven days of their scheduled appointment, although the latter effect could also be the result of other temporal changes. Other strategies that have been proposed to improve linkage to and retention in care include decentralization of HIV services [30, 31], incorporation of HIV care into primary care programs [32], eliminating requirements for additional pre-therapy counseling visit [33], and provision of transportation stipends to minimize patient costs of care [34]. Given the complex and population-specific array of factors contributing to poor retention rates across the continent, it is likely that a combination of interventions will be required to overcome the widespread epidemic of program loss in sub-Saharan Africa [35].

Our findings also provide support for use of patient-centered, mHealth-based applications to improve health care delivery in similar settings, for which prior efficacy data is limited [36]. The intervention studied here is among the first patient-centered, mHealth intervention to improve patient-provider clinical communication for HIV care in a resource-limited setting. In contrast, prior mHealth evaluations for HIV care delivery have either relied on health care workers as the end users, as opposed to patients themselves [27, 37], or focused on notifications to improve adherence [18, 19, 38]. Two characteristics of our combination intervention should be considered for future mHealth interventions for low-literacy end users. First, as described in the Methods section, we designed the intervention in response to a conceptual framework explaining the health problem of interest. Preliminary work identified a combination of poor communication and financial constraints as primary barriers to return to clinic after abnormal test results, and our combination intervention leveraged the regional mobile health infrastructure and a transportation reimbursement to address both simultaneously.

The second factor supporting success of the intervention was use of a co-creation model of development, through partnership with local stakeholders (that is, clinical staff) and targeted end users, during application design, evaluation, and implementation [22]. Important lessons were derived from this process, which undoubtedly prevented a substantial proportion of communication failures. For example, pre-intervention surveys revealed that patients at the clinic overwhelmingly supported an SMS-based system to improve clinical communication, but also that an important minority were concerned about breaches in confidentiality. In response to this input, we selected a randomized design of three message formats, interchanging clarity through direct messages with privacy through coded and PIN-protected messages. While piloting the intervention, we also learned that use of the number “1” is restricted on many Ugandan mobile phones, and so we removed this option from all PIN numbers and response functions during the intervention stage.

Our study is subject to a number of limitations. First, we implemented an unblinded, non-randomized study design, and found differences in the study periods in participant age and proportion naïve to ART. While the differences in time to clinic return and time to ART initiation could be explained by unmeasured confounders or temporal changes in clinic outcomes, we believe this is unlikely because 1) our results were independent of the most likely confounding variables, including immune status, gender, and education, 2) our effect sizes were large and demonstrated across all randomized subgroups, and 3) the intervention period immediately followed the control period and no other changes in clinical protocols took place during either period. Second, our study was implemented within a research setting. Effectiveness studies should be pursued to evaluate its impact on a larger, real-world scale. In its current format, our intervention requires a single phone and/or Internet connection, a staff member to process and enter results, and the cost of a transportation stipend ($6 per patient in our case). These inputs are comparable to the cost, infrastructure, and human resources requirements for standard point-of-care CD4 tests, which require similar resources and cost approximately $10 per sample. While the greatest threat to sustainability of our intervention is the cost of the transportation reimbursement, it is likely that this could be a one-time cost for most patients. Data from our site and others has demonstrated that economic restoration is rapid and largely complete during the first year of ART therapy, suggesting the potential to decrease reliance on economic support early after initiation of care [39, 40]. While not implemented in our study design, the automation of financial incentives could be an important addition to this platform with the use of a “mobile money” platform (that is, automatic transfer of money through a mobile phone account). Lastly, we developed and tested a combination intervention; thus, we cannot assess whether the impact we detected was a result of either intervention alone or the combination of the two. However, we developed the combination intervention in response to a conceptual framework derived from formative research noting both financial and communication barriers to care, and thus posited that both were needed to meaningfully impact care. For example, a transportation reimbursement for early clinic return would be of little benefit in the absence of a notification system to alert patients of the need for early return. An important area of future study will be to identify the independent effects of the SMS messaging component with and without a transportation stipend.

## Conclusions

In summary, we found significant improvements in time to clinic return and ART initiation after abnormal laboratory results in southwestern Uganda with an SMS-based combination intervention including laboratory result notifications and transportation reimbursements. The benefit of the intervention was seen in three different SMS formats with varying degrees of clarity and confidentiality. Future investigations should evaluate the scalability of similar interventions. Successful reproducibility would add a promising strategy to improving HIV care in sub-Saharan Africa.

## Additional files

Additional file 1: Table S1. Characteristics of study participants in each SMS message arm with abnormal CD4+ T-lymphocyte results. Additional file 2: Table S2. Characteristics and time to clinic return for study participants with normal CD4+ T-lymphocyte results.

## Abbreviations

ART: Antiretroviral therapy
mHealth: Mobile Health
PIN: Personal identification number
SMS: Short messaging service
