# Supplementary material for: A combination SMS and transportation reimbursement intervention to improve HIV care following abnormal CD4 test results in rural Uganda: a prospective observational cohort study
Source: BMC Med. 2015 Jul 6;13:160. doi: 10.1186/s12916-015-0397-1 (PMC4494725; doi:10.1186/s12916-015-0397-1)
Supplement: Additional file 1: Table S1. — Characteristics of study participants in each SMS message arm with abnormal CD4+ T-lymphocyte results. [file 12916_2015_397_MOESM1_ESM.doc]

**Additional file 1: Table S1. Characteristics of study participants with abnormal CD4+ T-lymphocyte results**

|  | Direct SMS Message Group  (n = 46) | PIN SMS Message Group  (n = 49) | Coded SMS Message Group  (n = 43) | *P*-value |
| --- | --- | --- | --- | --- |
| Female Gender (n, %) | 24 (52) | 26 (53) | 25 (58) | 0.83 |
| Age (median, IQR) | 31 (27 – 40) | 30 (26 – 36) | 30 (25 – 37) | 0.41 |
| Education (n, %) |  |  |  | 0.67 |
| <Primary | 3 (7) | 5 (10) | 4 (9) |  |
| Any primary | 23 (50) | 29 (59) | 19 (44) |  |
| Any secondary | 13 (28) | 9 (18) | 15 (35) |  |
| >Secondary | 7 (15) | 6 (12) | 5 (12) |  |
| ART Naïve (n, %) | 39 (85) | 37 (76) | 34 (79) | 0.43 |
| Mbarara Resident (n, %) | 29 (63) | 26 (53) | 28 (65) | 0.44 |
| Days from enrollment until laboratory result, median (IQR) | 10 (9 – 16) | 10 (7 – 14) | 11 (9 – 16) | 0.19 |
| Clinician-specified abnormal CD4 result threshold, median (IQR) | 350 (350 – 350) | 350 (350 – 350) | 350 (350 – 350) | 0.85 |
| CD4 Result, median (IQR) | 198 (115 – 276) | 226 (91 – 307) | 230 (137 – 282) | 0.87 |

SMS: short-message service text message

PIN: Personal identification number

IQR: Inter-quartile range

ART: antiretroviral therapy
