# Supplementary material for: A combination SMS and transportation reimbursement intervention to improve HIV care following abnormal CD4 test results in rural Uganda: a prospective observational cohort study
Source: BMC Med. 2015 Jul 6;13:160. doi: 10.1186/s12916-015-0397-1 (PMC4494725; doi:10.1186/s12916-015-0397-1)
Supplement: Additional file 2: Table S2. — Characteristics and time to clinic return for study participants with normal CD4+ T-lymphocyte results. [file 12916_2015_397_MOESM2_ESM.doc]

Additional file 2: Table S2. Characteristics and time to clinic return for study participants with normal CD4+ T-lymphocyte results

|  | Control Group  (No SMS Message)  (n = 91) | Intervention Group  (Normal SMS Message)  (n = 247) | *P*-value |
| --- | --- | --- | --- |
| Female Gender (n, %) | 53 (58) | 176 (71) | 0.02 |
| Age (median, IQR) | 37 (30 – 43) | 34 (26 – 40) | 0.03 |
| Education (n, %) |  |  | 0.31 |
| <Primary | 3 (5) | 31 (13) |  |
| Any primary | 30 (50) | 126 (51) |  |
| Any secondary | 21 (35) | 67 (27) |  |
| >Secondary | 6 (10) | 23 (9) |  |
| ART Naïve (n, %) | 33 (36) | 197 (80) | <0.001 |
| Mbarara Resident (n, %) | 61 (69) | 154 (62) | 0.24 |
| Proportion returning to clinic on the next scheduled visit date (n, %) | 53 (58) | 156 (63) | 0.41 |
| Proportion returning to clinic within seven days of the next scheduled visit date (n, %) | 56 (62) | 180 (73) | 0.04 |

SMS: short-message service text message

PIN: Personal identification number

IQR: Inter-quartile range

ART: antiretroviral therapy
